# Supplementary material for: Histological, Transcriptomic, and Functional Analyses Reveal the Role of Gibberellin in Bulbil Development in Lilium lancifolium
Source: Plants (Basel). 2024 Oct 24;13(21):2965. doi: 10.3390/plants13212965 (PMC11547782; doi:10.3390/plants13212965)
Supplement: Supplementary file 1 [file plants-13-02965-s001.zip › plants-3228278-supplementary.pdf]

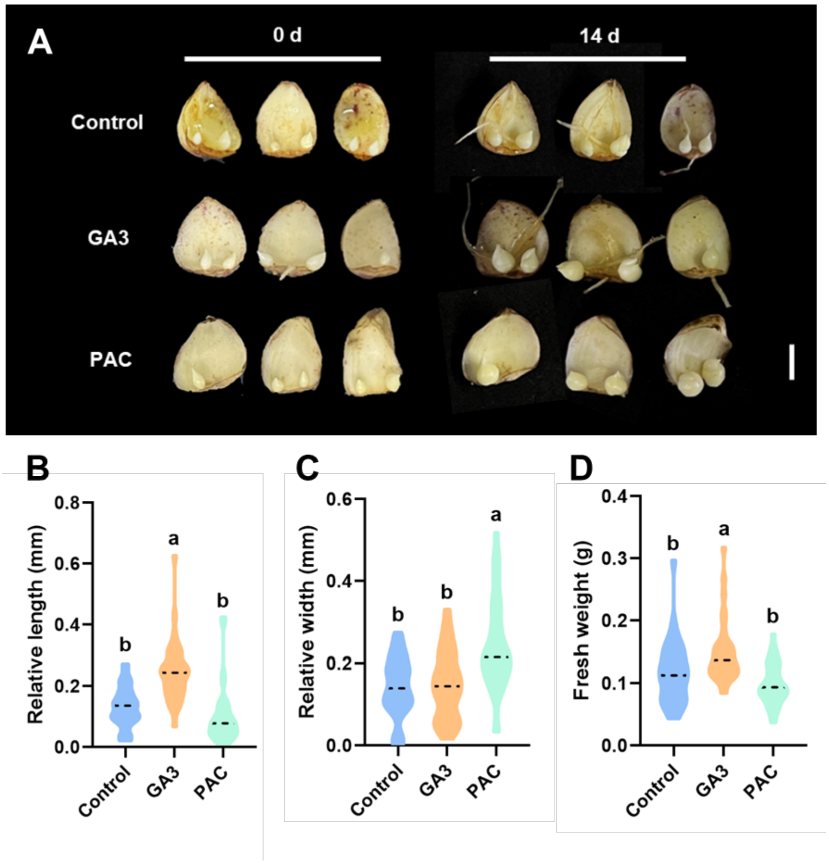

Figure S1. Gibberellin promote bulblet development by modulating cell length. A. Phenotypes of bulblet development with the mock, GA_3 and PAC treatment. 30 independent scales were used for each treatment. Scale bar represents 1 cm. **B-D.** Relative length (B), relative width (C) and fresh weight of bulblet with the mock, GA_3 and PAC treatment. The relative length or width was delineated as the discrepancy between the bulblet diameter at 14 d and the bulblet diameter at 0 d. 60 independent scales under control, GA_3 and PAC treatment groups were used for calculation. Student's *t*-test was used for statistical analysis in panels B-D (*: $P < 0.05$; **: $P < 0.01$).

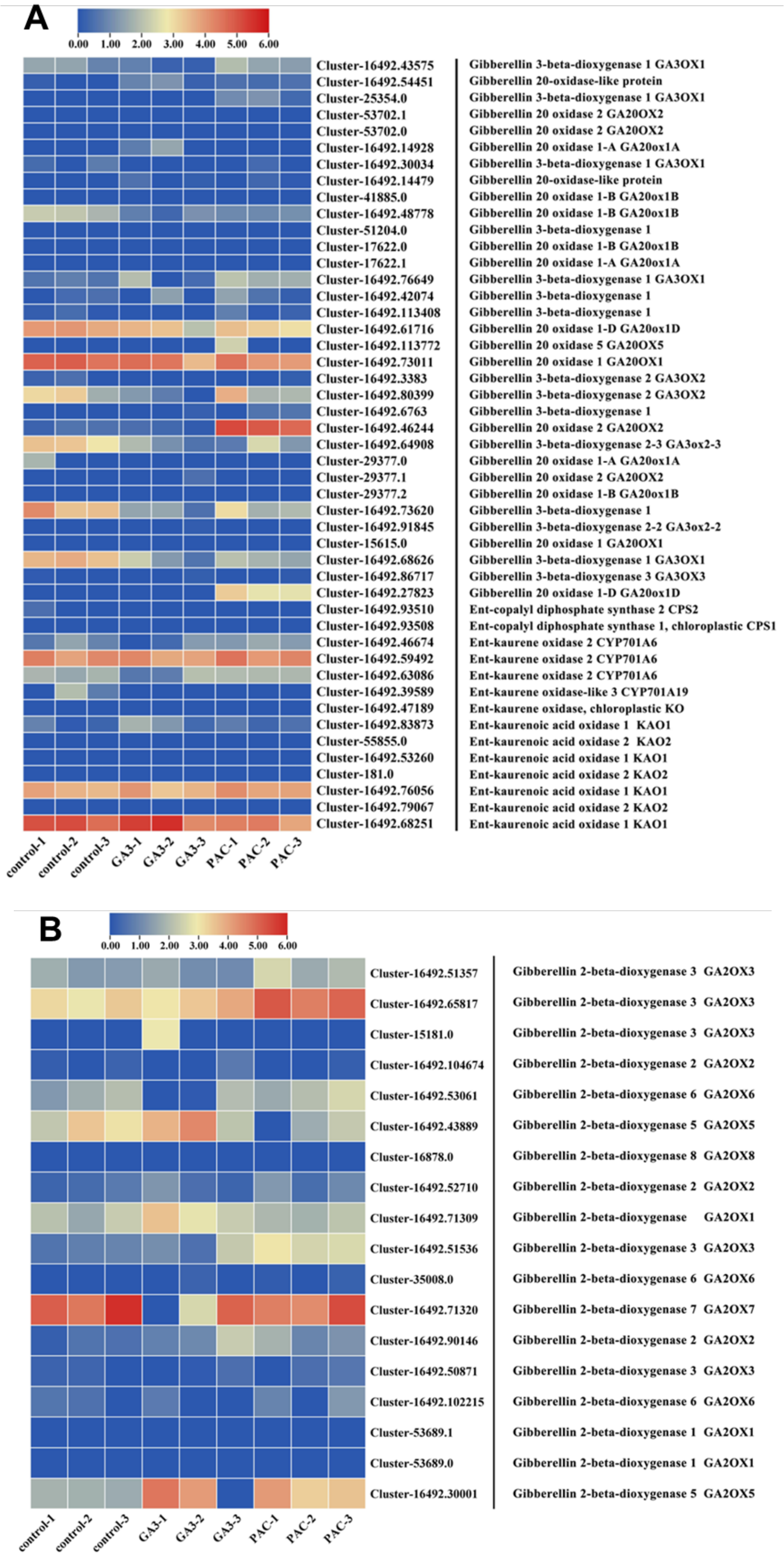

Figure S2. Heat map showing expression patterns of gibberellin synthesis and metabolism pathway genes under control, GA_3 and PAC treatment at S2 stage. The color scale from blue to red represents the FPKM value from low to high.

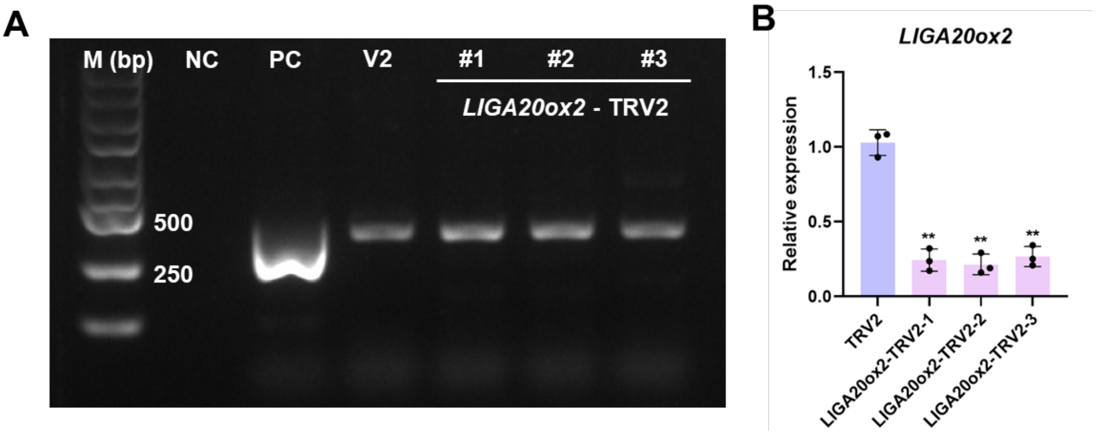

Figure S3. Validation of *LlGA20ox2*-TRV2 and TRV2 plants. A. Detection of the virus COAT PROTEIN fragments in the bulbils of TRV2 (V2) and *LlGA20ox2*-TRV2 plants using RT-PCR with extracted RNA. M: DNA marker; NC: non-agroinfiltrated plants used as the negative control; PC: recombinant pTRV2 plasmids with target gene specific fragment were used as positive controls. **B.** Relative expression of *LlGA20ox2* in the bulblets of TRV2 and *LlGA20ox2*-TRV2 plants, verified by RT-qPCR. The data represents mean ± s.d. of three biological replicates. Student' s *t*-test was used for statistical analysis (*: $P < 0.05$; **: $P < 0.01$).

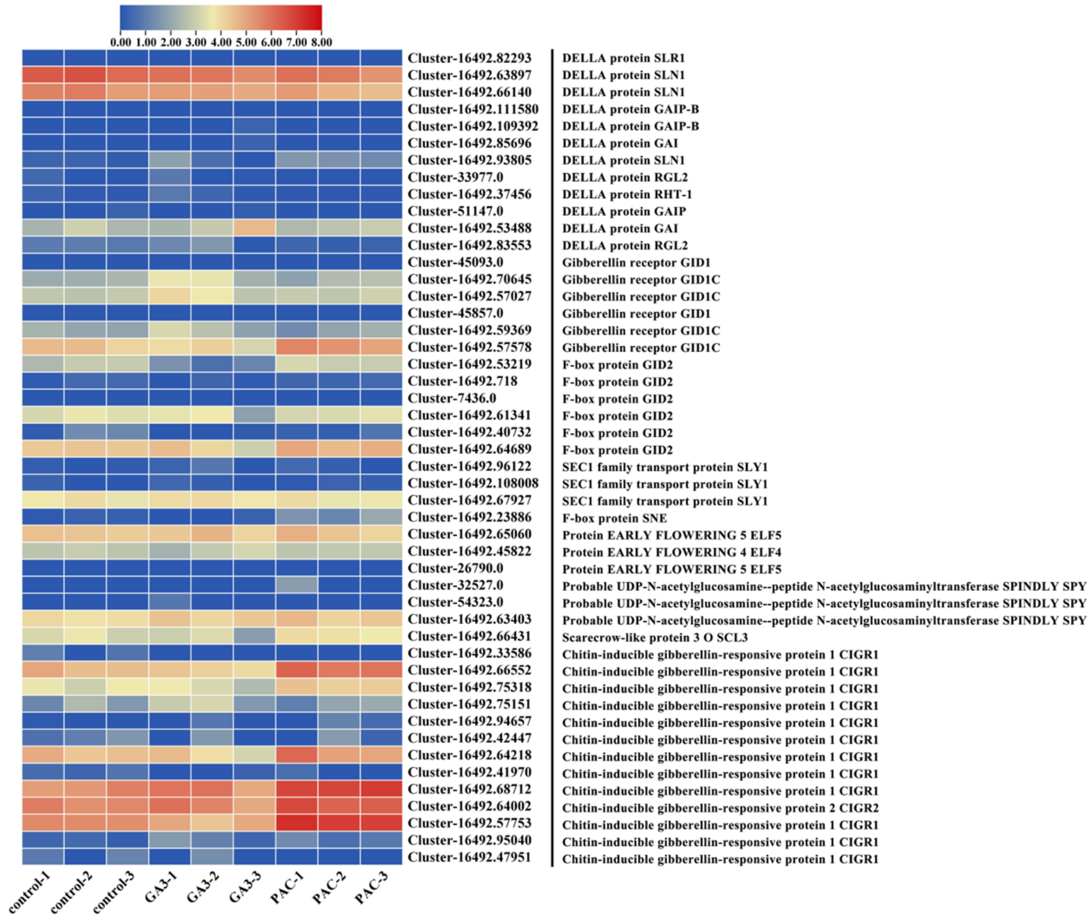

Figure S4. Heat map showing expression patterns of gibberellin signaling pathway genes under control, GA_3 and PAC treatment at S2 stage. The color scale from blue to red represents the FPKM value from low to high.

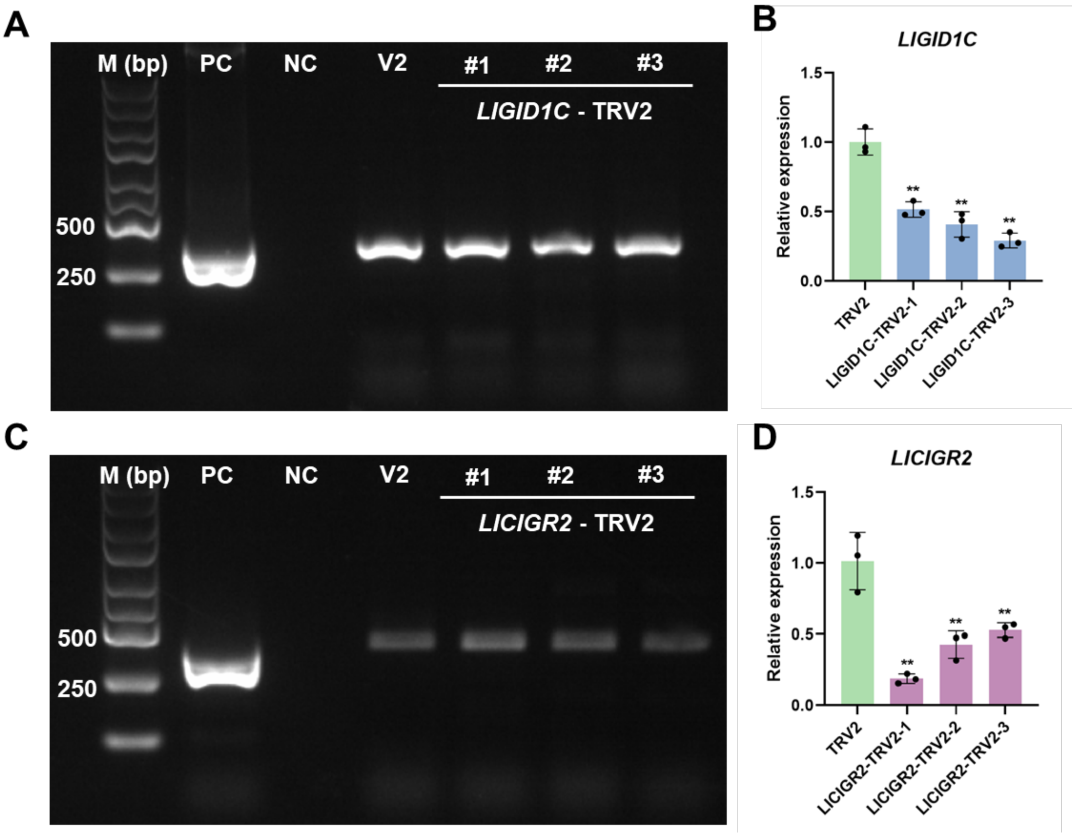

Figure S5. Validation of *LlGID1C*-TRV2, *LlCIGR2*-TRV2 and TRV2 plants. A. Detection of the virus COAT PROTEIN fragments in the bulbils of TRV2 (V2) and *LlGID1C*-TRV2 plants using RT-PCR with extracted RNA. M: DNA marker; NC: non-agroinfiltrated plants used as the negative control; PC: recombinant pTRV2 plasmids with target gene specific fragment were used as positive controls. **B.** Relative expression of *LlGID1C* in the bulblets of TRV2 and *LlGID1C*-TRV2 plants, verified by RT-qPCR. The data represents mean ± s.d. of three biological replicates. **C.** Detection of the virus COAT PROTEIN fragments in the bulbils of TRV2 (V2) and *LlCIGR2*-TRV2 plants using RT-PCR with extracted RNA. **D.** Relative expression of *LlCIGR2* in the bulblets of TRV2 and *LlCIGR2*-TRV2 plants, verified by RT-qPCR. The data represents mean ± s.d. of three biological replicates. Student' s *t*-test was used for statistical analysis (*: P < 0.05; **: P < 0.01).

Table S1. The primer sequences used in this study

ID	Sequence (5’- 3’)
Primer sequences of RT-qPCR	
FP-F	TCGCCTACATCGCTAACC
FP-R	TTCCCAATAATCGCAAGACC
Cluster-16492.74233_F (LlGA20ox2)	GGTCCTCCTCCTCCCGACTC
Cluster-16492.74233_R	TCCGTCGAGGAAGCCTTGGA
Cluster-16492.46655_F (LlGID1C)	TCTGTAACGGCGCTGTCGTC
Cluster-16492.46655_R	ACATGGTGGGCGATGTTCCC
Cluster-16492.65752_F (LlCIGR2)	TGCCTGAGATGGAAGGCGAT
Cluster-16492.65752_R	CTGCGTAACCACCGGCTGTC
Primer sequences of VIGS	
LlGA20ox2-TRV2-F	gtgagtaaggttaccgaattcCCGCCATTGACGAACTTGA
LlGA20ox2 -TRV2-R	tggaggccttctagagaattcTGCTGCCAGGCTTCCGTC
LlGID1C-TRV2-F	gtgagtaaggttaccgaattcATGGCTGTGCCACTCAACACA
LlGID1C-TRV2-R	tggaggccttctagagaattcGGCGGAGAGCGGGGCCTG
LlCIGR2-TRV2-F	gtgagtaaggttaccgaattcACCCTGAAGACGGATCACAGTT
LlCIGR2-TRV2-R	tggaggccttctagagaattcTGTGTTTCAGTTCATTCGGGTCT
Primer sequences of gene overexpression	
LlGA20ox2-eGFP-F	gcagcggccgaattccccgggATGAATTCAAGCCCTACTTCGG
LlGA20ox2-eGFP -R	cttgcatgcctgcagcccgggTTAGCAAATAGGGTTGCTGCCG
Primer sequences of yeast one-hybrid (Y1H) system	
LlGA20ox2-AbAi-F	cttgaattcgagctcggtaccAAGTCTGGCGAGGTATTTATAGGG
LlGA20ox2-AbAi-R	gtcgacagatccccgggtaccCACATTGAGGTCGTAGTTAGATCCA
LlAGL2-AD-F	gtaccagattacgctcatatgATGGGGAGAGGAAAGATTGAGAT
LlAGL2-AD-R	acgattcatctgcagctcgagTTATCCAAGTTGGAGTGCAGTCTG
